# Experimental Investigation on Behavior of Rectangular Concrete-Filled Tubular Columns Considering Diaphragms

**DOI:** 10.3390/ma13194412

**Published:** 2020-10-03

**Authors:** Sang-Lyul Cha, Jung-Soo Lee, Chan-Kyu Park, Jin-Keun Kim, Seung-Hee Kwon

**Affiliations:** 1 Department of Civil and Environmental Engineering, Korea Advanced Institute of Science and Technology, 291 Daehak-ro, Yuseong-gu, Daejeon 34141, Korea; maikuraki@kaist.ac.kr (S.-L.C.); concrete1@kaist.ac.kr (J.-K.K.); 2 Department of Civil and Environmental Engineering, Myungji University, 116 Myongji-ro, Cheoin-gu, Yongin-si 17058, Korea; jung86ss@gmail.com; 3 Institute of Consruction Technology, Samsung C & T Corporation, 26, Sanil-ro 6-gil, Gangdong-gu, Seoul 05288, Korea; helpme@samsung.com

**Keywords:** CFT column, compressive strength, failure behavior, diaphragm

## Abstract

Concrete-filled tubular (CFT) columns have been widely used as structural members because CFT columns synergize the advantages of steel and concrete resulting in high strength, high ductility, and large energy dissipation capacity. Numerous studies have been performed to understand the behavior of CFT columns. However, the behavior of CFT columns remains uncertain due to their inelastic behavior and uncertain confinement effects, especially when failure occurs. In addition, diaphragms, which are generally installed, make it more complicated to understand the behavior of CFT columns. The purpose of this study is to investigate the effects of the diaphragms on the failure behavior of the CFT columns. To this end, eighteen rectangular CFT columns were tested with five different loading cases. The experimental results suggest that the size of the diaphragm has significant effects on the compressive strength and toughness of the CFT columns. In order to facilitate the proper composite actions of steel and concrete, the size of a diaphragm has to be at least three-quarters of the cross-sectional area.

## 1. Introduction

Concrete-filled tubular (CFT) columns combine both the advantages of steel and concrete resulting in high strength, high ductility, and large energy dissipation capacity. These features arise from composite action between concrete and steel: Inner concrete prevents inward local bucking of steel while outer steel increase the compressive strength of concrete by confining lateral expansion of concrete [1]. In addition, steel casting can be used as a formwork for concrete, reducing the construction cost. CFT columns have therefore been widely used as structural members, especially in high-rise buildings [2].

Numerous studies have been performed to understand the behavior of CFT columns, by means of experimental and analytical approaches. O’Shea and Bridge developed design methods that can be used to estimate the strength of circular thin-walled CFT columns under different loading conditions considering small eccentricities [3]. Giakoumelis and Lam experimentally studied the strength of circular CFT columns considering steel tube thickness, concrete strength, and bonding conditions, and concluded that design codes of CFT columns underestimated the strength [4]. Zeghiche and Chaoui evaluated the effects of slenderness, load eccentricity, and compressive strength of concrete on load carrying capacity and strength of CFT columns in their study where 27 CFT columns were tested [5]. Studies considering the effects of diaphragms have also been performed because a diaphragm is generally installed to transmit efficiently the force and prevent from deformation of beam–column joint due to the applied external force [6]. Kwon et al. studied the long-term behavior of circular and rectangular CFT columns under central axial loading and concluded that diaphragm size did not affect the long-term behavior of CFT columns when diaphragm covers more than a half of the cross-sectional area of the inner concrete [7,8]. Vulcu et al. developed design approaches for steel beam-to-rectangular CFT column joints considering external diaphragms [9,10]. Zhang et al. presented seismic behavior of steel beam-to-circular CFT column assemblies with external diaphragms. They reported the ductility of CFT columns were affected by failure modes, axial loads, and constraint of steel tubes [11]. Wang and Lee investigated the behaviors of circular CFT columns considering the mixed diaphragms, and suggested the method to calculate an optimal mix of diaphragms [12]. Dong et al. proposed a bearing capacity calculation method for rectangular CFT columns with different internal construction characteristics [13]. They investigated the effects of longitudinal stiffeners, horizontal tie bars, studs, internal diaphragms, and steel reinforcement cages on the mechanical performance of CFT columns. They did not consider the size of the internal diaphragms.

Despite these numerous research endeavors, the behavior of CFT columns remains uncertain due to its inelastic behavior and uncertain confinement effects, especially when failure occurs. In particular, there is a hole in the middle for pouring concrete in the case of through-type or inner diaphragm, which is installed inside the CFT, and it inevitably influences the composite behavior of the steel tube and concrete and the distribution of force [14]. In addition, as the failure behavior of CFT columns can be affected by long-term deformation (i.e., creep), which causes stress redistribution between concrete and steel tube [15], the stress distribution of CFT columns is greatly affected by the size and shape of the diaphragm. Therefore, the effects of size of through-type or inner diaphragms on the behavior of CFT columns should be investigated in order to understand the behavior of the CFT columns or develop design codes.

The aim of this study is to investigate the failure behavior of CFT column, in particular, to explore the effects of the inner diaphragm size on the behavior of CFT columns after long-term deformation, which is sufficient to induce stress redistribution. To this end, experiments were conducted on 18 rectangular CFT columns under five different loading cases, and the experimental results were analyzed in detail.

## 2. Experimental Program

### 2.1. Specimens

**Figure 1:**
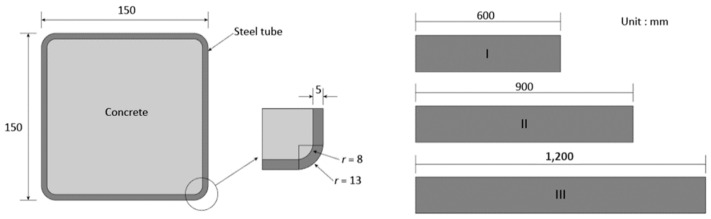
Dimensions of concrete-filled tubular (CFT) column specimens.

Figure 1 shows the dimensions of CFT column specimens. All the CFT columns have the same cross-sectional area: concrete is 140 × 140 mm and the thickness of the steel tube is 5 mm. In order to manufacture the steel tube, steel plates were cut and bent with a steel bending machine. The bent steel plates were connected by continuous welding along the longitudinal direction according to ASTM A1085. To minimize development of residual stresses, the steel tubes were heat treated. Concrete was cast in the steel tube to manufacture the CFT columns. The exposed surfaces of concrete were covered by wet curing cloth to prevent the evaporation of water. Two specimens were manufactured for each variable.

**Figure 2:**
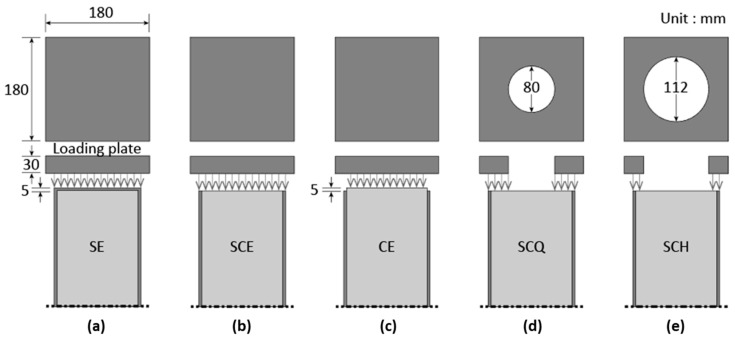
Loading cases, (**a**) SE; (**b**) SCE; (**c**) CE; (**d**) SCQ; (**e**) SCH.

In order to investigate the effects of diaphragms on the behavior of CFT columns, five loading cases were employed and are presented in Figure 2 (a) only steel tube is loaded (SE), (b) concrete and steel tube are both loaded (SCE), (c) only concrete is loaded (CE), (d) 3/4 area of concrete and steel tube are loaded (SCQ), and (e) half of concrete and steel tube are loaded (SCH). In addition, the specimens for SCQ and SCH were manufactured in three different lengths to study the effects of specimen length (600, 900, and 1200 mm, see Figure 1) on the behavior of CFT columns. Table 1 summarizes the number of the specimens.

### 2.2. Material Properties

Mix proportion of concrete is shown in Table 2. The water-to-binder ratio was 0.32, and type I cement, which is designated for general use was used according to ASTM C150. Aggregate of 15 mm maximum size was used considering workability and specimen dimensions. Cylindrical concrete specimens of ф 150 mm × 300 mm were tested to determine the concrete properties using universal testing machine (UTM) according to ASTM C39 and C469. A load cell, three linear variable displacement transducers (LVDTs), and a dial gauge were used to measure the load, vertical displacement, and lateral displacement, respectively. The compressive strength and the elastic modulus were 47.7 MPa and 27.1 GPa, respectively, after 28 days. The Poisson’s ratio of concrete was 0.18.

Standard steel coupons were also tested to determine the properties of the steel tube using UTM according to ASTM E8. A load cell and an extensometer were used to measure the load and displacement. The elastic modulus and the yield strength of steel were 196 GPa and 284 MPa respectively. Poisson’s ratio of steel provided by a supplier was 0.30. Table 3 summarizes the mechanical properties of concrete and steel.

### 2.3. Test Setup and Instrumentation

**Figure 3:**
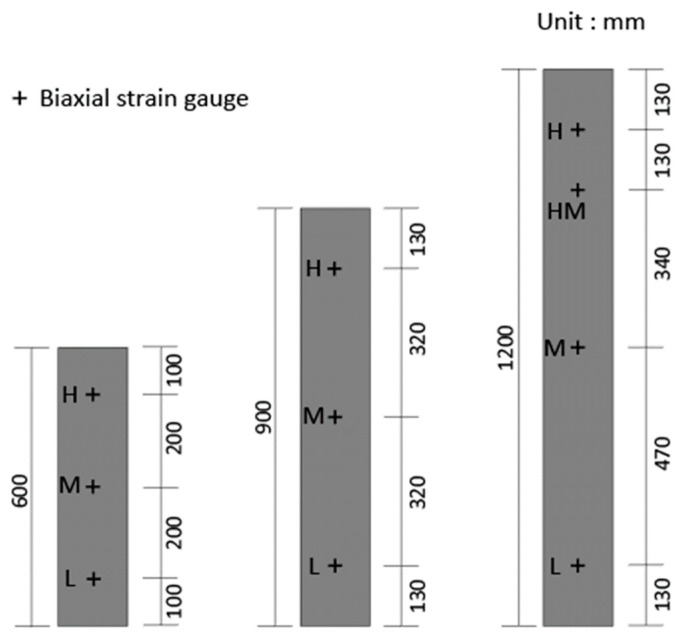
Location of steel strain gauges.

**Figure 4:**
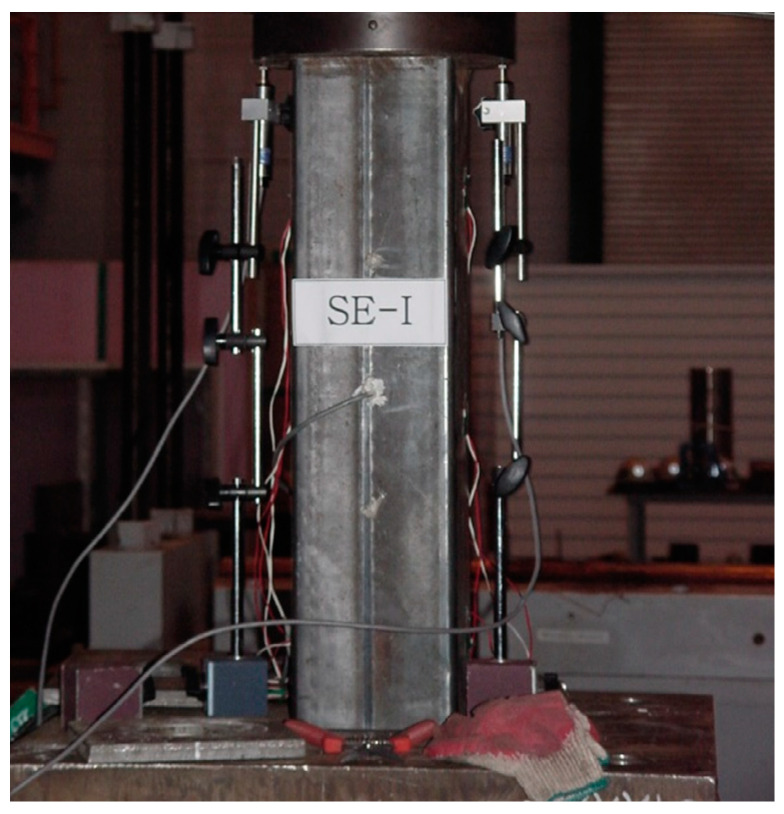
Test setup for compressive strength of CFT columns.

The embedded strain gauges were positioned at the center of the CFT columns with the length of 600 and 900 mm to measure the deformation of concrete. In case of the CFT columns with the length of 1200 mm, the embedded strain gauges were located at three different positions (two strain gauges at 130 mm from the ends, one strain gauge in the middle). Steel strain gauges were also installed at the outer surface of the steel tube as shown in Figure 3. In case of the CFT columns with the length of 600 and 900 mm, the gauges were installed at three points, while the strains of the steel tube were measured at four points for the CFT columns with the length of 1200 mm. Biaxial strain gauges were attached at each point to measure the longitudinal and lateral strain. In addition, two LVDTs were used to measure the axial shortening of the CFT columns as shown in Figure 4. 

All the CFT columns were under sustained loads for 100 days to consider the stress redistribution due to creep. After the sustained loads, the CFT columns were loaded at a crosshead displacement ratio of 0.001 mm/s using UTM with a loading capacity of 2000 kN until the failure. All the experimental data were simultaneously obtained by means of a data acquisition system.

## 3. Results and Discussions

### 3.1. General Behavior

**Figure 5:**
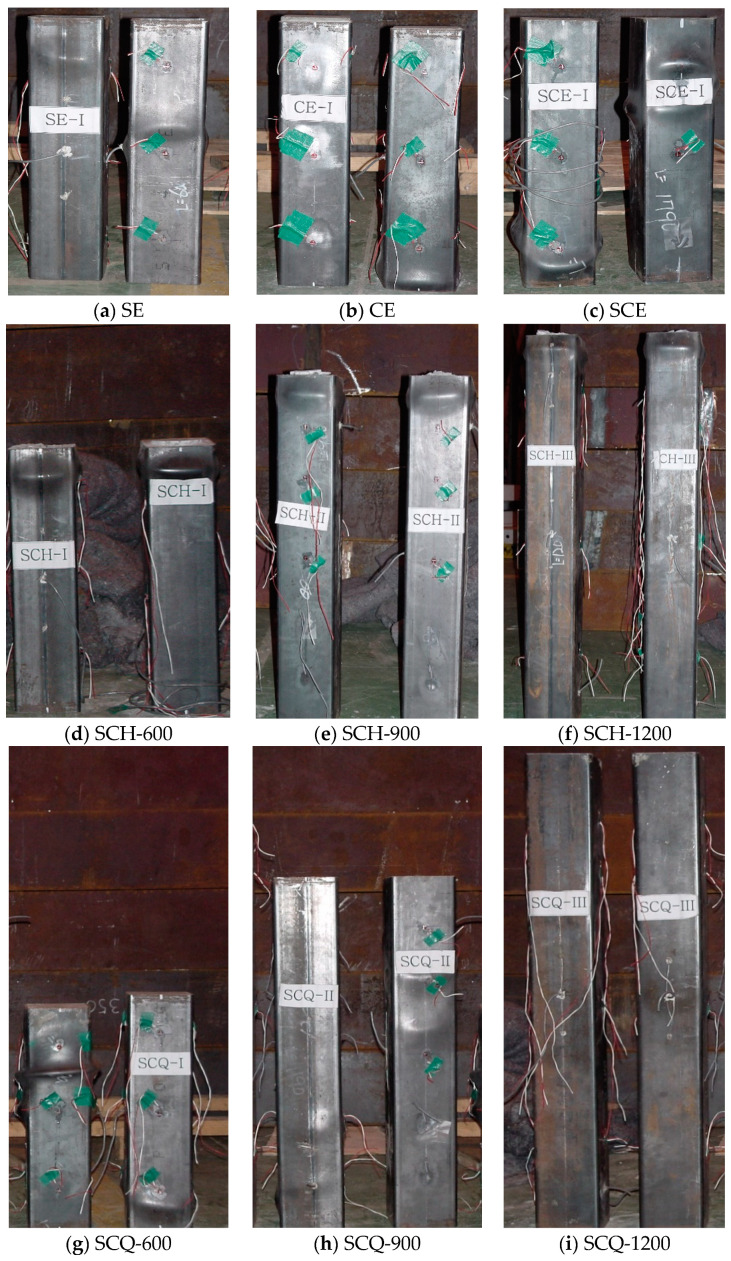
Failure mode of CFT columns, (**a**) SE; (**b**) CE; (**c**) SCE; (**d**) SCH-600; (**e**) SCH-900; (**f**) SCH-1200; (**g**) SCQ-600; (**h**) SCQ-900; (**i**) SCQ-1200.

The CFT column specimens were axially loaded to investigate failure behavior of CFT columns according to the size of the diaphragm and column length, and the experimental results are summarized in Table 4. Note that the experimental data for SCQ-600–2 and SCQ-900–2 are omitted due to data loss. Figure 5 shows the CFT column specimens after tests. For all the specimens, there were no noticeable changes on the surface of the specimens before the ultimate stress has developed. After the ultimate stress, local buckling bulges appeared on the surface of the specimens. As the stress increased, the existing local buckling bulges grew and new buckling bulges were observed. Generally, 1–3 buckling bulges were observed in the longitudinal direction of the CFT columns during the tests. 

### 3.2. Loading Case SE

**Figure 6:**
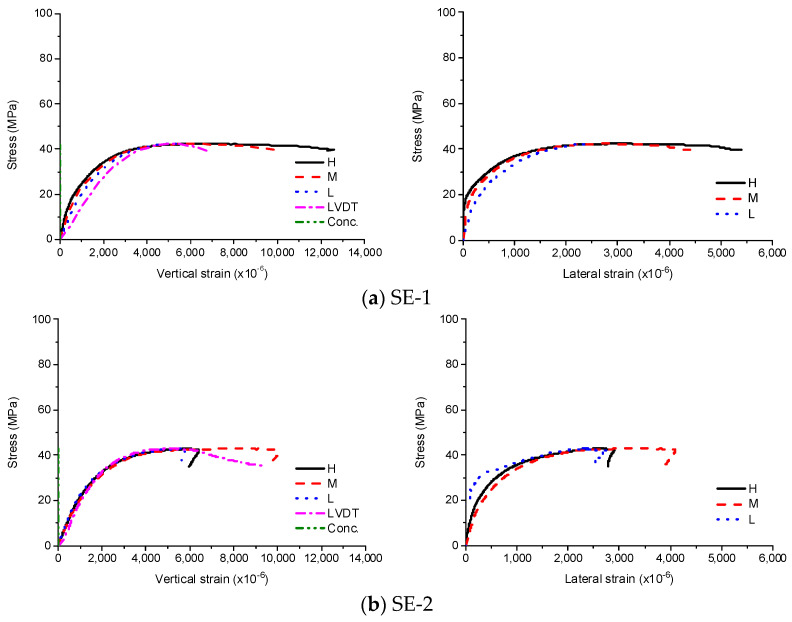
Experimental results for SE, (**a**) SE-1; (**b**) SE-2.

As the loads applied to the steel tube were not transferred to the interior concrete, whose strain was close to zero during the experiments, the experimental results shown in Figure 6 were identical to that of thin hollow cylinder of steel. Compared to the steel coupons, the stress–strain curve is rounded and shows a decreasing elastic modulus with increasing stress, because the diameter-to-thickness ratio is small resulting in plastic buckling failure [16,17,18,19]. After reaching the maximum stress, both specimens showed outward elephant’s foot buckling occurring at the mid-height or one end of the specimen (high-height). As plastic buckling is accompanied by a decrease in the stiffness due to wrinkles, there was no notable yield point. The stress–strain curves were similar regardless of the location until the ultimate stress. After the ultimate stress has been reached, vertical and lateral strains where local buckling occurred significantly increased. In case of SE-1, the local buckling occurred at the multiple regions while the local buckling occurred at mid-height for SE-2. The average compressive strength and the corresponding strain were 42.6 MPa and 5060 × 10^−6^, respectively.

### 3.3. Loading Case CE

**Figure 7:**
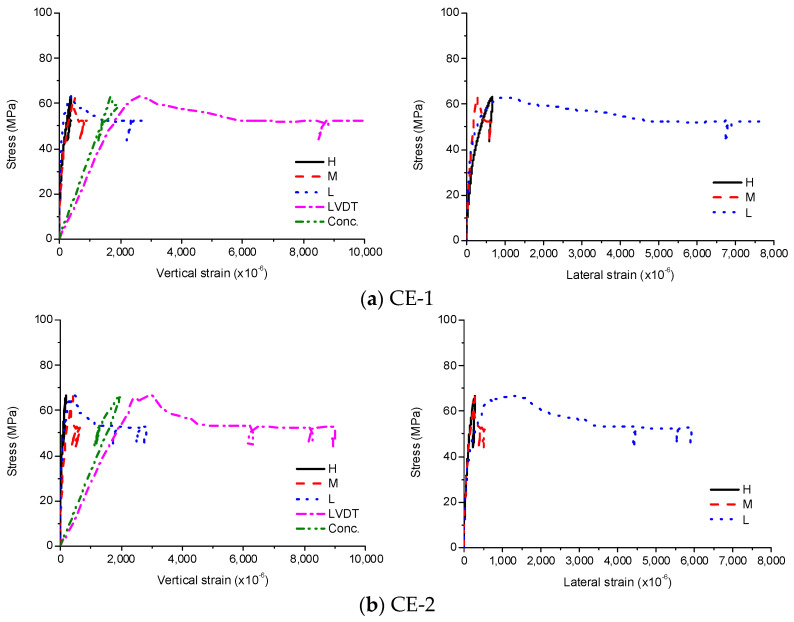
Experimental results for CE, (**a**) CE-1; (**b**) CE-2.

The experimental results for CE are presented in Figure 7. As the loads were only applied to concrete and, therefore, not transferred to the steel tube at low stress levels, it was only the concrete that initially resisted the load. As the stress grew, cracking occurred in the concrete and the lateral displacement of the concrete increased. Therefore, the concrete was confined by the steel tube, increasing both vertical and lateral deformation of the steel tube. After reaching the ultimate stress, the concrete was severely damaged, so the embedded strain gauges could not measure the vertical strain and local buckling of the steel tube occurred at the bottom. However, when the sudden stress drop occurred due to the concrete failure, the stress was recovered immediately by the increase of confinement showing yielding behavior. The stress remained above 50 MPa even when the vertical strain was more than 8000 × 10^−6^. The average compressive strength of CE was 65.0 MPa and the corresponding strain was 2780 × 10^−6^. 

### 3.4. Loading Case SCE

**Figure 8:**
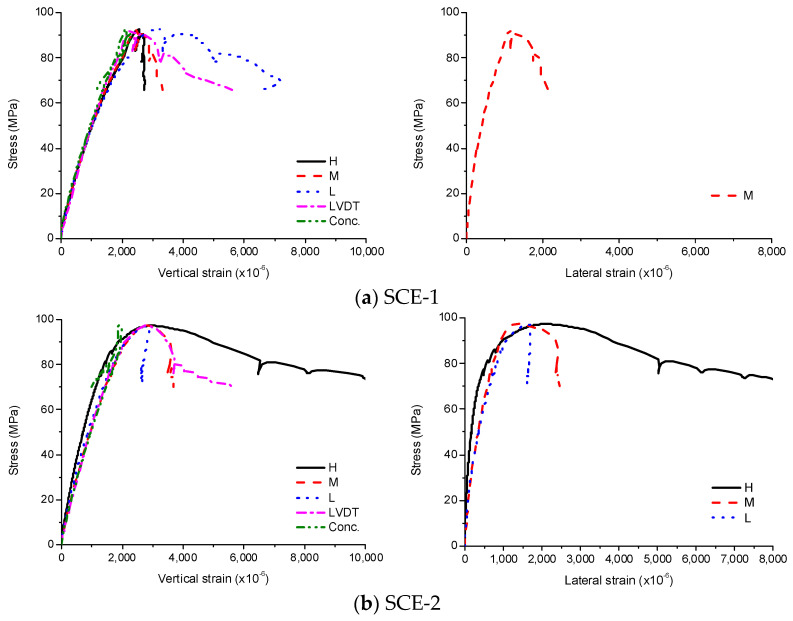
Experimental results for SCE, (**a**) SCE-1; (**b**) SCE-2.

As concrete and steel tube both resisted the external loads, the vertical strains of steel tube and concrete were almost identical before the local buckling occurred as shown in Figure 8. The stress–strain curve was linear initially; as the stress developed, it became nonlinear due to cracking in the interior concrete. After the maximum stress, the local buckling occurred due to the concrete failure and the stress decreased. The stress rapidly decreased as soon as the buckling occurred, resulting in a rapid increase in the vertical and lateral strains of the steel tube, simultaneously the rate decreased because the lateral deformation of the concrete was restrained by the steel tube. As it has been the case for CE, the sudden drop of stress occurred due to the concrete failure, and the stress was recovered immediately because the lateral deformation of the concrete was more restrained by the steel tube, increasing the confinement effects. The average compressive strength of SCE was 95.2 MPa and the corresponding strain was 2570 × 10^−6^.

### 3.5. Loading Case SCH

**Figure 9:**
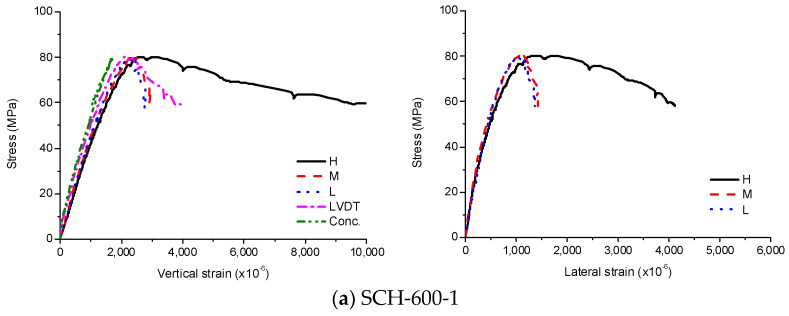

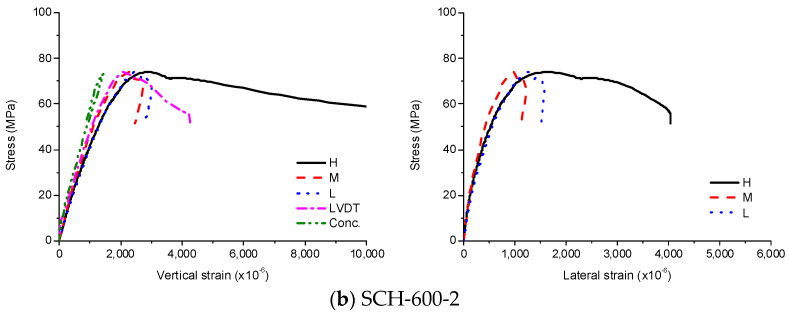
Experimental results for SCH-600, (**a**) SCH-600–1; (**b**) SCH-600–2.

**Figure 10:**
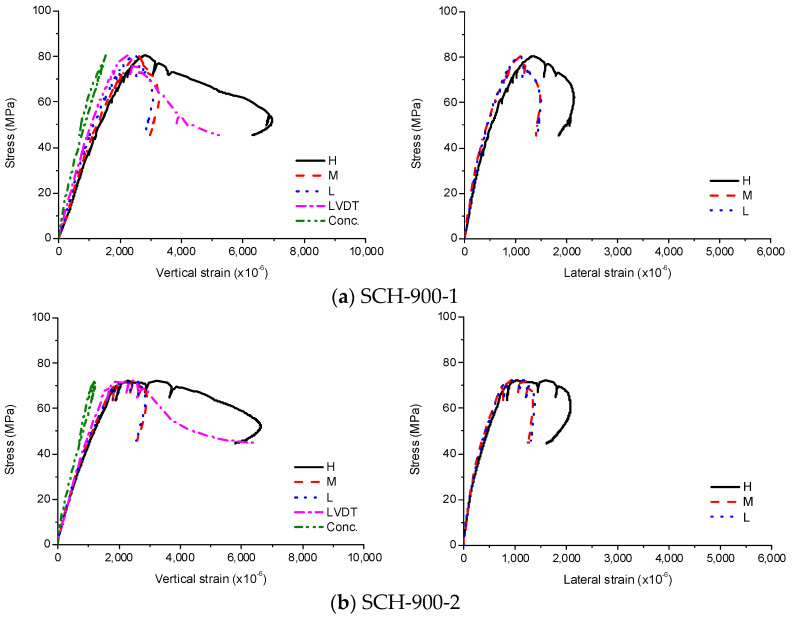
Experimental results for SCH-900, (**a**) SCH-900–1; (**b**) SCH -900–2.

**Figure 11:**
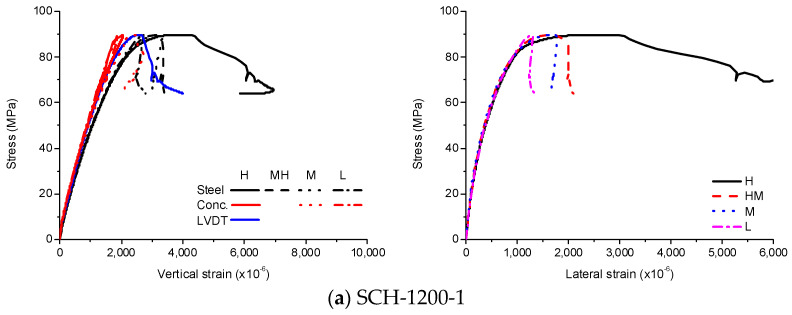

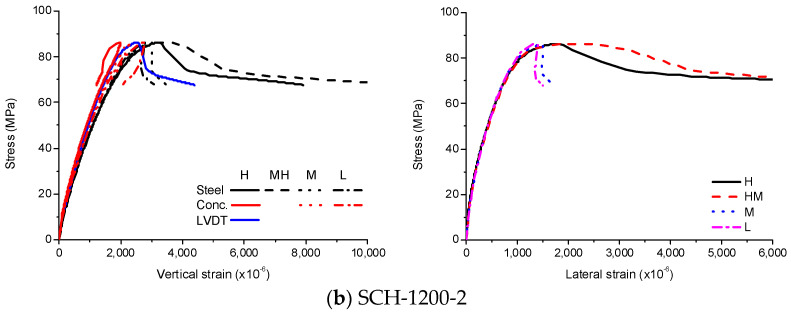
Experimental results for SCH-1200, (**a**) SCH-1200–1; (**b**) SCH-1200–2.

**Figure 12:**
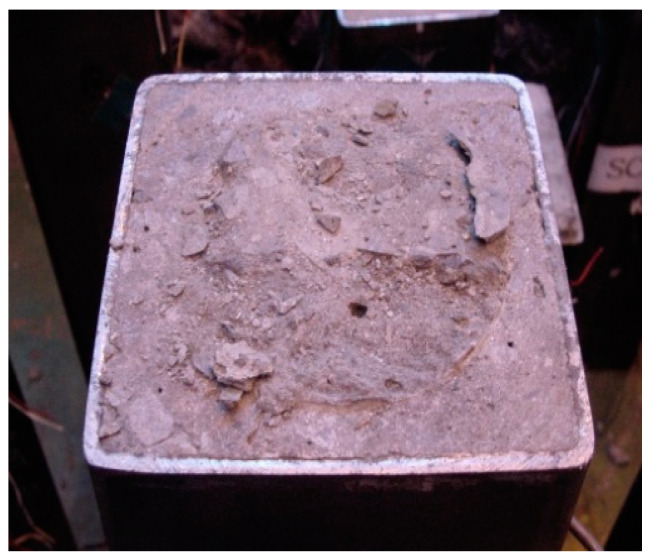
Bearing failure in concrete for SCH.

The stress-strain curves of SCH are shown in Figure 9, Figure 10 and Figure 11. As similar to the other loading cases, the stress-strain curves for these specimens were linear at low stress levels and became non-linear at high stress levels. Before the ultimate stress, there were no noticeable differences in the stress-strain curves according to the length of the CFT columns. Therefore, the ultimate stress and the peak strain were similar regardless of the length of the CFT columns. After the ultimate stress, the local buckling occurred at the high-height, because the stress in the concrete near the loading plate was much higher than that in the central concrete due to the holes in the loading plate. The higher stress caused the bearing failure of the concrete as shown in Figure 12. Therefore, the vertical strain and lateral strains of the steel tube were significantly different depending on the concrete failure. The vertical strain measured by the embedded strain gauge was similar to the strain measured by LVDT before the cracking in concrete occurred. After the cracking in concrete occurred, the vertical strain measured by the embedded strain gauge, which was located at the mid-height, was smaller than the strain measured by LVDT, because the bearing failure in the concrete affects the measurement at the top end only. The average compressive strength was 77 MPa, and the corresponding strain was 2140 × 10^−6^.

### 3.6. Loading Case SCQ

**Figure 13:**
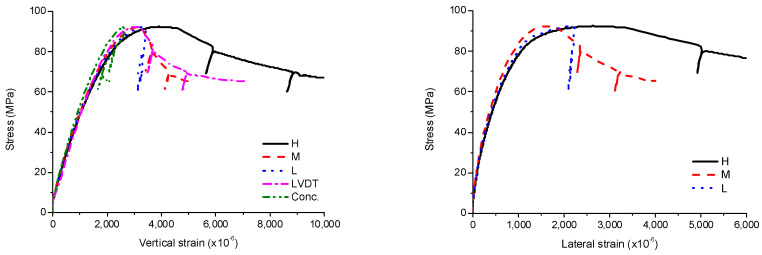
Experimental results for SCQ-600–1.

**Figure 14:**
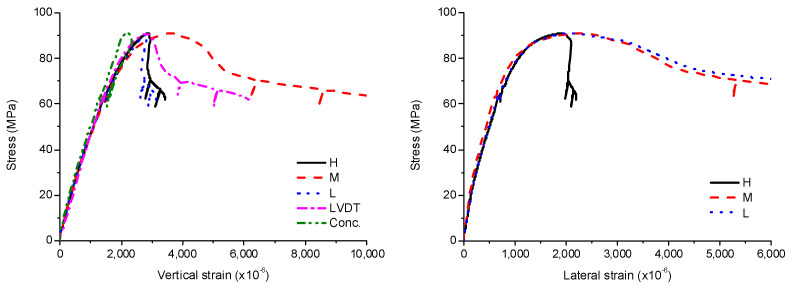
Experimental results for SCQ-900–1.

**Figure 15:**
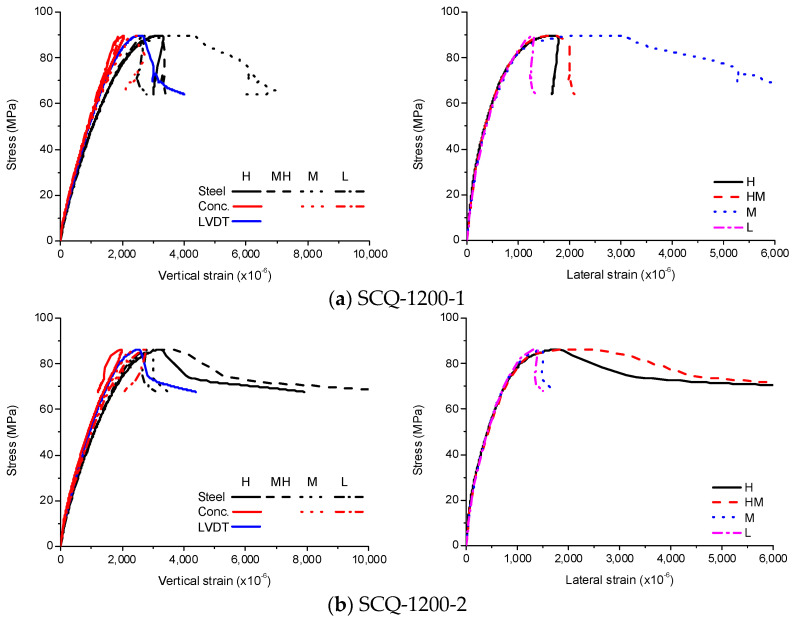
Experimental results for SCQ-1200, (**a**) SCQ-1200–1; (**b**) SCQ-1200–2.

The stress–strain curves of SCH are shown in Figure 13, Figure 14 and Figure 15. The experimental results for SCQ -600–2 and SCQ -900–2 were omitted due to data loss. As similar to SCE and SCH, the stress–strain curves of SCQ were initially linear and became nonlinear with increasing stress. After the ultimate stress, the local buckling occurred resulting in noticeable generation of buckling bulges at multiple regions. In case of the specimens with the length of 1200 mm, the local and global buckling simultaneously occurred. Unlike SCH, there was no bearing failure because the load was well-distributed generating lower stresses in the concrete near the load-bearing plate. The compressive strength and the corresponding strain decreased with the increase of the length, but the difference was marginal. The average compressive strength was 90 MPa, showing values similar to that of SCE specimens, and the corresponding strain was 2730 × 10^−6^.

### 3.7. The Effects of Diaphragms

#### 3.7.1. Average of Experimental Results

In order to investigate the effects of the diaphragms on the failure behavior of the CFT columns, experimental results were averaged for comparisons by employing the method proposed by Zhao et al. [20].

**Figure 16:**
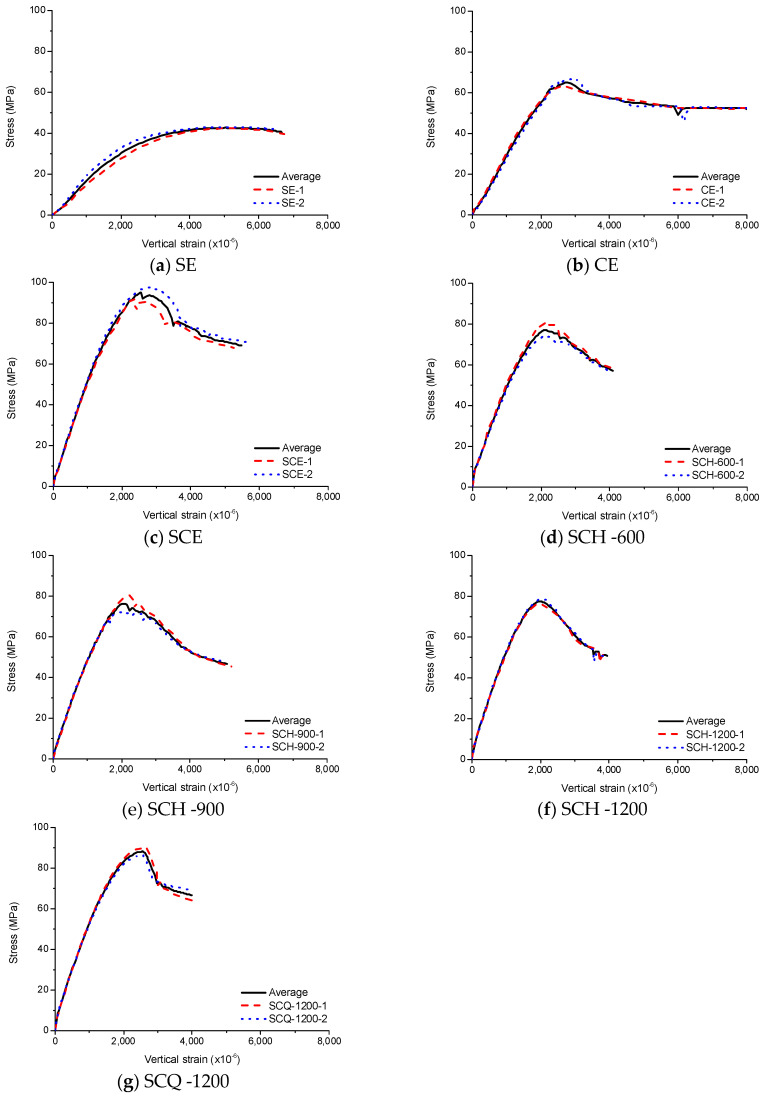
Experimental results for SCQ-1,200, (**a**) SE; (**b**) CE; (**c**) SCE; (**d**) SCH-600; (**e**) SCH-900; (**f**) SCH-1200; (**g**) SCQ-1200.

There are about 1000 data points for each specimen. One data point was selected for every 10 data points. The value averaged from the sequential three data points was taken to eliminate the effect of the fluctuation on the measurement. Fifty equally spaced values were selected from the zero point to the peak point and from the peak point to the end point. The end point was chosen to have the same length between the peak point and the end point. The data points corresponding to 100 values were calculated by interpolation of the data points. Therefore, the data points for each comparison specimen can be averaged using 100 interpolated data points. The results are shown in Figure 16.

#### 3.7.2. Comparison of Experimental Results

**Figure 17:**
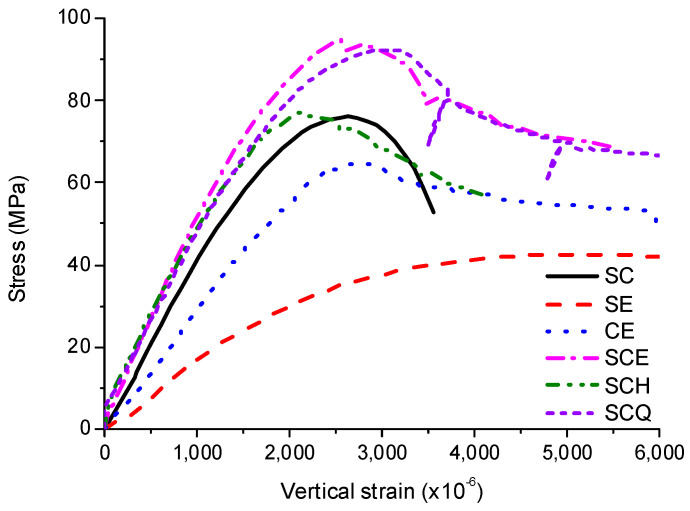
Comparison of averaged experimental results for the specimen with 600 mm length.

Figure 17 shows the averaged stress–strain curves for CFT columns with 600 mm length and SC which is the sum of SE and concrete. As expected, compressive strengths of SCE and SE were the highest and lowest, respectively. The compressive strengths of SCE and SCQ were similar, and those of SCH and SC were similar. As CE was under the similar condition to confined concrete, the compressive strength of CE was much higher than that of concrete. As the steel tube only resists vertical loads induced by the friction, the compressive strength of CE was smaller than that of SC. Although the vertical load was transferred to steel tube by the diaphragm in case of SCH, the compressive strength of SCH was similar to SC due to concrete failure induced by stress concentration near ends. The compressive strengths of SCE and SCQ were much higher than that of SC because the compressive strength of concrete was increased by the confinement.

As concrete only resists the vertical loads at a low stress level for CE and the vertical loads were resisted by only the steel tube for SE, the elastic modulus was smaller than the other CFT columns. Therefore, the stress–strain curves were almost the same for all the CFT columns except for CE and SE up to 40 MPa in the region where the stress and strain are in linear relationship. This relationship changed to non-linear at high stress levels. Although the peak strain occurred at ~2000–3000 × 10^−6^ similar to concrete [21], the behavior of the CFT columns was different from concrete after the peak strain. Concrete generally shows sudden failure after the peak strain, while most of the CFT columns showed ductile behavior. After the peak strain, the vertical stress decreased rapidly and became nearly constant for SCE, SCQ, and CE. However, the stress of SCH decreased continuously and became lower than that of CE after 3800 × 10^−6^. This means that the size of the diaphragm has a significant effect on toughness which is the energy absorption capacity.

**Figure 18:**
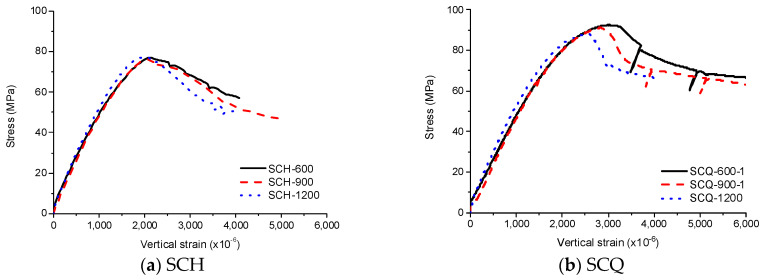
Comparison of the averaged experimental results with different length, (**a**) SCH; (**b**) SCQ.

**Figure 19:**
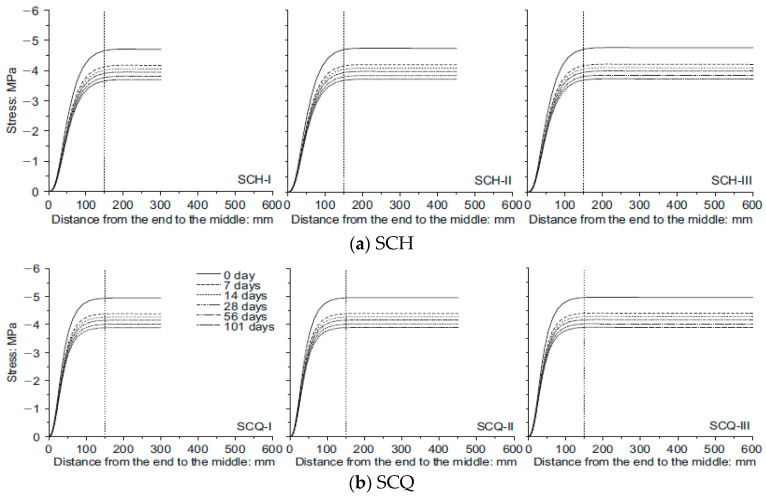
Vertical stress at the center of the concrete [8], (**a**) SCH; (**b**) SCQ.

**Figure 20:**
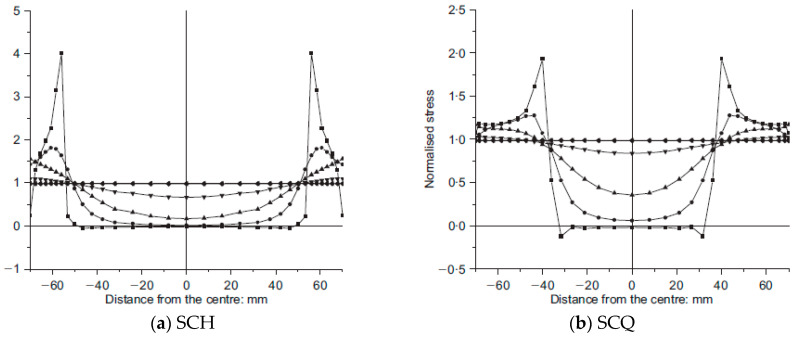
Normalized vertical stress distribution over cross-section of CFT columns [8], (**a**) SCH; (**b**) SCQ.

The averaged stress–strain curves for SCH and SCQ according to the CFT column length are shown in Figure 18. Instead of the averaged SCQ-600 and SCQ-900, SCQ-600–1 and SCQ-900–1 are presented in Figure 18b due to the data loss. The stress–strain curves of SCH were similar to each other, while the stress–strain curves of SCQ were different. The compressive strength decreased with the length increase due to global buckling induced by manufacturing defects, residual stresses, inaccurate test set-up, etc. Therefore, most of them failed at the near mid-height. However, as the failure of the CFT columns were greatly affected by stress concentration near ends of the CFT columns in case of SCH due to small size of the diaphragm, the local buckling occurred at the near ends regardless of the CFT column length. Figure 19 and Figure 20 show the stress in central concrete according to the longitudinal direction [8]. The stress concentration decreased with the increase of the depth from the ends and the stress over the cross section became uniform over the depth of 150 mm following Saint-Venant’s principle [22]. The stress distribution of the concrete does not depend on the length of the specimen. It depends on the diaphragm size. The stress observed in SCH was 4 times greater than the uniform stress at the ends, while the stress developed in SCQ was twice larger. Therefore, it can be concluded that the compressive strength is significantly affected by the size of the diaphragms.

## 4. Conclusions

In order to investigate the behavior of CFT columns, uniaxial compression test was conducted using 18 CFT columns. In particular, there were five loading cases and three different lengths of the CFT columns to understand the effects of the diaphragms and the length on the behavior of CFT columns. The following conclusions can be drawn.

1. The size of the diaphragms has a significant effect on the strength of the CFT columns. The compressive strength of SCE was the highest, followed by SCQ, SCH, CE, and SE. In order to ensure the proper composite actions of steel and concrete, the size of a diaphragm has to be at least three-quarters of the cross-sectional area.
2. The compressive strength and toughness of CE was greatly increased due to the confinement effects by the steel tube.
3. When half of the cross-sectional area of the concrete was covered with the diaphragm (SCH), CFT columns failed due to the stress concentration resulting in compressive strength similar to the case when only concrete is loaded (CE).
4. When three-quarters of the cross-sectional area of the concrete was covered with the diaphragm (SCQ), the compressive strength and toughness of the CFT columns were similar to those of the CFT columns whose the cross-sectional area was entirely covered by the diaphragm (SCE).
5. There were no noticeable differences in the strength and toughness according to the length of the SCH because of the concrete failure near ends induced by stress concentration. On the other hand, the compressive strength of SCQ decreased as the length of the specimen increased due to global buckling at mid-height induced by manufacturing defects, residual stresses, and inaccurate test set-up etc.

## Figures and Tables

**Table 1:** Number of CFT columns.

Loading Case	CFT Column Length		
	600 mm	900 mm	1200 mm
SE	2	-	-
SCE	2	-	-
CE	2	-	-
SCQ	2	2	2
SCH	2	2	2

**Table 2:** Mix proportion of concrete.

W/B	Unit Weight (kg/m^3^)					
	W	C	FA ^1^	S	G	SP ^2^
0.32	172	480	60	780	1000	7.2

^1^ Fly ash ^2^ Super plasticizer.

**Table 3:** Mechanical properties of concrete and steel [8].

Material	Elastic Modulus (GPa)	Strength (MPa)	Poisson’s Ratio
Concrete	27.1	47.7 (28 days)	0.18
Steel	196	284 (Yielding)	0.30

**Table 4:** Summary of the experimental results.

Title 1	Compressive Strength (MPa)	Peak Strain (× 10^−6^)	Failure Mode
SE-1	42.3	5150	Local buckling at multiple regions
SE-2	42.8	4970	Local buckling in near mid-height
CE-1	63.1	2610	Failure of concrete near bottom
CE-2	66.8	2950	Failure of concrete near bottom
SCE-1	92.9	2390	Local buckling near bottom
SCE-2	97.4	2740	Local buckling at multiple regions
SCH-600–1	80.3	2110	Local buckling near top
SCH-600–2	74.1	2140	Local buckling near top
SCH-900–1	80.4	2170	Local buckling near top
SCH-900–2	72.4	2460	Local buckling near top
SCH-1200–1	76.1	1940	Local buckling near top
SCH-1200–2	78.7	2000	Local buckling near top
SCQ-600–1	92.5	3020	Local buckling near mid-height
SCQ-600–2	Data loss	Data loss	Local buckling near bottom
SCQ-900–1	91.1	2810	Local buckling at multiple regions
SCQ-900–2	Data loss	Data loss	Local buckling at multiple regions
SCQ-1200–1	89.8	2580	Local and global buckling
SCQ-1200–2	86.3	2500	Local and global buckling
